# Functional reorganization of intranetwork and internetwork connectivity in patients with Ménière’s disease

**DOI:** 10.1038/s41598-023-44090-x

**Published:** 2023-10-05

**Authors:** Jing Li, Yangming Leng, Hui Ma, Fan Yang, Bo Liu, Wenliang Fan

**Affiliations:** 1 Department of Radiology, Union Hospital, Tongji Medical College, Huazhong University of Science and Technology, Wuhan, 430022 China; 2 Hubei Province Key Laboratory of Molecular Imaging, Wuhan, 430022 China; 3 Department of Otorhinolaryngology Head and Neck Surgery, Union Hospital, Tongji Medical College, Huazhong University of Science and Technology, Wuhan, 430022 China

**Keywords:** Neurological disorders, Prognostic markers, Network models

## Abstract

Ménière’s disease (MD) is associated with functional reorganization not only in the auditory or sensory cortex but also in other control and cognitive areas. In this study, we examined intranetwork and internetwork connectivity differences between 55 MD patients and 70 healthy controls (HC) in 9 well-defined resting-state networks. Functional connectivity degree was lower in MD compared to HC in 19 brain areas involved in the somatomotor, auditory, ventral attention, default mode, limbic, and deep gray matter networks. In addition, we observed lower intranetwork connectivity in the auditory, ventral attention, and limbic networks, as well as lower internetwork connectivity between the somatomotor and limbic networks, and between the auditory and somatomotor, deep gray matter, and ventral attention networks, and between the deep gray matter and default mode network. Furthermore, we identified 81 pairs of brain areas with significant differences in functional connectivity between MD patients and HC at the edge level. Notably, the left amygdala’s functional connectivity degree was positively correlated with MD’s disease stage, and the ventral attention network’s intranetwork connectivity was positively correlated with the healthy side vestibular ratio. Our findings suggest that these functional network reorganization alterations may serve as potential biomarkers for predicting clinical progression, evaluating disease severity, and gaining a better understanding of MD’s pathophysiology. Large-scale network studies using neuroimaging techniques can provide additional insights into the underlying mechanisms of MD.

## Introduction

Ménière’s disease (MD) is an inner ear disorder characterized by spontaneous episodic vertigo, fluctuating sensorineural hearing loss, tinnitus, and aural, which may potentially lead to a significant decline in one’s quality of life^1,2^. MD most commonly starts between the ages of 40 and 60 years, and the prevalence of MD are variable, from 3.5 to 513 cases per 100,000^1,3,4^. Endolymphatic hydrops (EH) has been observed as the most classic pathogenesis in MD patients^5^, which was potentially caused by disturbed fluid homeostasis^1^. Advancements in MRI techniques, particularly gadolinium chelate enhancement, have enabled clinicians to non-invasively visualize various aspects of the inner ear’s structure and function^6^. In addition, MD patients have exhibited reduced hippocampal volume in high resolution structural MRI scans, which correlates significantly with the severity of hearing and vestibular function on the affected side^7,8^. This suggests that MD may impact not only the inner ear but also other areas of the brain. Furthermore, other studies have noted a reduction in fractional anisotropy and an increase in apparent diffusion coefficient of the vestibulocochlear nerve in MD patients compared to healthy controls^9^. Although MRI studies of MD have been extensive, the current research primarily focuses on changes in brain structure, leaving the corresponding alterations in brain function still largely unexplored.

Resting-state functional magnetic resonance imaging (rs-fMRI) is a widely utilized tool for evaluating brain function, as it enables the identification of spontaneous neural activity associated with self-initiated behavior and detects intrinsic functional networks in the brain^10–12^. Interestingly, while rs-fMRI has been extensively employed in studying brain function, relative few studies have directly examined MD itself. Instead, research has primarily focused on analogous conditions that share MD-like symptoms, providing insights into abnormal brain network organization. For instance, patients experiencing vertigo have shown reduced functional connectivity between the thalamus and visual cortex^13^, decreased connectivity between the left hippocampus and bilateral central opercular^14^, and reduced connectivity between the precuneus and left precentral gyrus^15^. Moreover, they exhibit decreased intranetwork functional connectivity in the right precuneus within the posterior default mode network^16^. Patients with sensorineural hearing loss have demonstrated functional reorganization not only within the auditory network^17,18^, but also in related brain areas involved in sensory processing, motor control, and cognitive functions^19–21^. Patients with tinnitus have exhibited abnormal functional brain network connectivity, not only within the auditory-limbic network^22^ but also across other networks, including the executive network, control network and default model network^23–25^. While these findings are valuable, they predominantly pertain to symptoms shared by MD and similar conditions. Given that MD patients experience similar symptoms, it raises the question of whether comparable functional reorganization of brain networks occurs in individuals with MD.

In this study, our objective was to explore the patterns of functional reorganization in intranetwork and internetwork connectivity between MD patients and healthy controls. Intranetwork connectivity refers to the strength of connections within specific functional brain networks^16,26,27^, representing ‘local’ interactions within a network. For instance, within the auditory network, regions responsible for processing sound signals are highly interconnected, facilitating efficient communication when processing auditory information. Conversely, internetwork connectivity pertains to interactions between different functional brain networks^16,26,27^, representing ‘global’ communication. For example, the auditory network may interact with the visual network when processing multisensory information.

We hypothesized that MD could potentially lead to reconfigurations not only within the auditory or sensory cortex but also in other areas associated with control and cognitive functions. To test this hypothesis, we examined the functional connectivity patterns of nine well-defined resting-state networks, including the auditory network (AUN), visual network (VSN), somatomotor network (SMN), dorsal attention network (DAN), ventral attention network (VAN), limbic network (LBN), frontoparietal network (FPN), default mode network (DMN), and deep gray matter network (DGN) at three different levels: integrity level, network level, and edge level.

## Results

### Clinical findings

We enrolled 55 patients with unilateral MD. The demographic characteristics of the included subjects are presented in Table 1. There was no significant difference in age (*p* = 0.443), sex (*p* = 0.988) and education level (*p* = 0.779) between the MD patients and the HC.

**Table 1:** Demographic and clinical variables.

Subjects	MD	HC	*P* value
Number of subjects	55	70	
Age (mean ± SD) years	52.0 ± 12.8	53.7 ± 12.0	0.443
Gender (male/female)	26/29	33/37	0.988
Education level (mean ± SD) years	10.6 ± 3.2	10.4 ± 3.9	0.779
Duration (mean ± SD) years	3.07 ± 4.71		
MD side (Left/Right)	32/23		
MD stage (I/II/III/IV)	4/9/34/8		
PTA of affected side	55.0 ± 17.8		
PTA of healthy side	17.0 ± 9.5		
VR of affected side	0.34 ± 0.07		
VR of healthy side	0.27 ± 0.05		
SP/AP of affected side	0.60 ± 0.39		
SP/AP of healthy side	0.27 ± 0.13		

A Pearson chi-squared test was used for gender comparison. independent samples t-tests were used for age, education comparisons.

*MD* Ménière’s disease, *HC* healthy control, *PTA* pure tone average, *SP* Summating potential, *AP*, action potential, *VR*: vestibular hydrops ratio.

For MD patients, twenty-six patients (47.3%) were male and 29 patients (52.7%) were female. The mean age of these patients are 52 years old (standard deviation: 12.8 years old). The duration of disease ranged from 1 month to 30 years (mean 3.07 years), with a median of 1.5 years (25th percentiles: 6 months; 75th percentiles: 3 years). Among them, 32 patients (58.2%) were left-sided affected and 23 patients (41.8%) were right-sided affected. Four patients (7.3%) were classified as MD stage I, 9 patients (16.4%) MD stage II, 34 patients (61.8%) stage III and 8 patient (14.5%) stage IV.

On affected side, no cochlear EH was found in 14 patients (25.5%), mild in 38 patients (69.1%), and significant in 3 patients (5.5%). The proportion of none, mild and significant EH in vestibular was the same as cochlear EH. As for non-affected side, cochlear EH was rated none in 48 patients (87.3%) and mild in 7 patients (12.7%). No vestibular EH was identified in 50 patients (90.9%) and mild in 5 patients (9.1%).

Thirty-seven patients underwent glycerol test. Twenty-one of them (56.76%) presented positive results and 16 (43.24%) negatives. ECochG was performed in 40 patients. Twenty-three of them (57.5%) exhibited positive results on affected side, 12 (30%) negative and no clear waveforms could be elicited in 5 patients (12.5%). Fifty-two patients underwent caloric test. Among them, 22 patients (42.31%) had normal results, 26 (50%) had abnormal CP value on MD affected side and 4 (7.69%) had bilateral vestibular hypofunction.

### Integrity level: altered node degree in patients with MD

**Figure 1:**
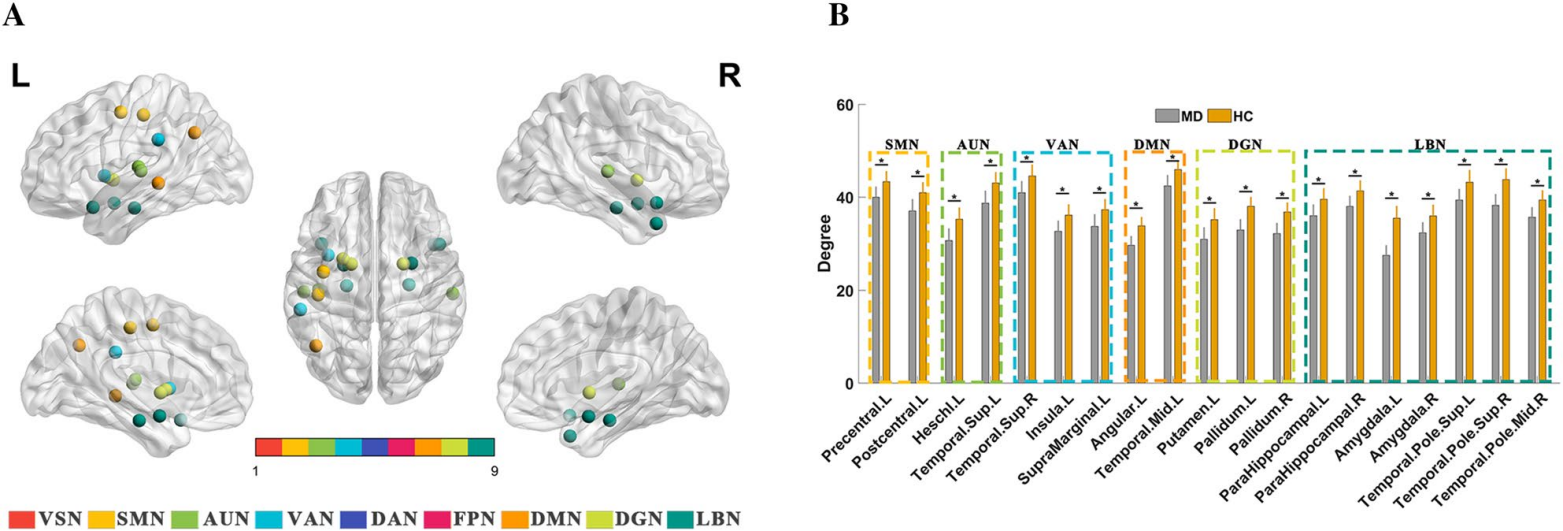
Altered node degree in patients with MD at the integrity level. (**A**) MD presented significantly (*p* < 0.05, FDR corrected) lower degree compared to HC in 19 brain regions (displayed on the brain surface). (**B**) The histogram shows the comparison between MD and HC of network degree. *VSN* visual network, *SMN* somatomotor network, *AUN* auditory network, *VAN* ventral attention network, *DAN* dorsal attention network, *FPN* frontoparietal network, *DMN* default mode network, *DGN*, deep gray matter network, *LBN*, limbic network, *MD*, Ménière’s disease, *HC*, healthy control, *indicates *p* < 0.05 after FDR correction.

Compared to HC, patients with MD showed lower node degree in 19 brain regions (*p* < 0.05, FDR corrected) belonging to SMN, AUN, VAN, DMN, DGN, and LBN, including the left precentral, left insula, bilateral parahippocampal, bilateral amygdala, left postcentral, left supramarginal, left angular, left putamen, bilateral pallidum, left Heschl, bilateral superior temporal (AAL regions 81 and 82), bilateral superior temporal pole (AAL regions 83 and 84), left middle temporal, and right middle temporal pole (Fig. 1A,B).

### Network level: altered intranetwork and internetwork connectivity strengths in patients with MD

**Figure 2:**
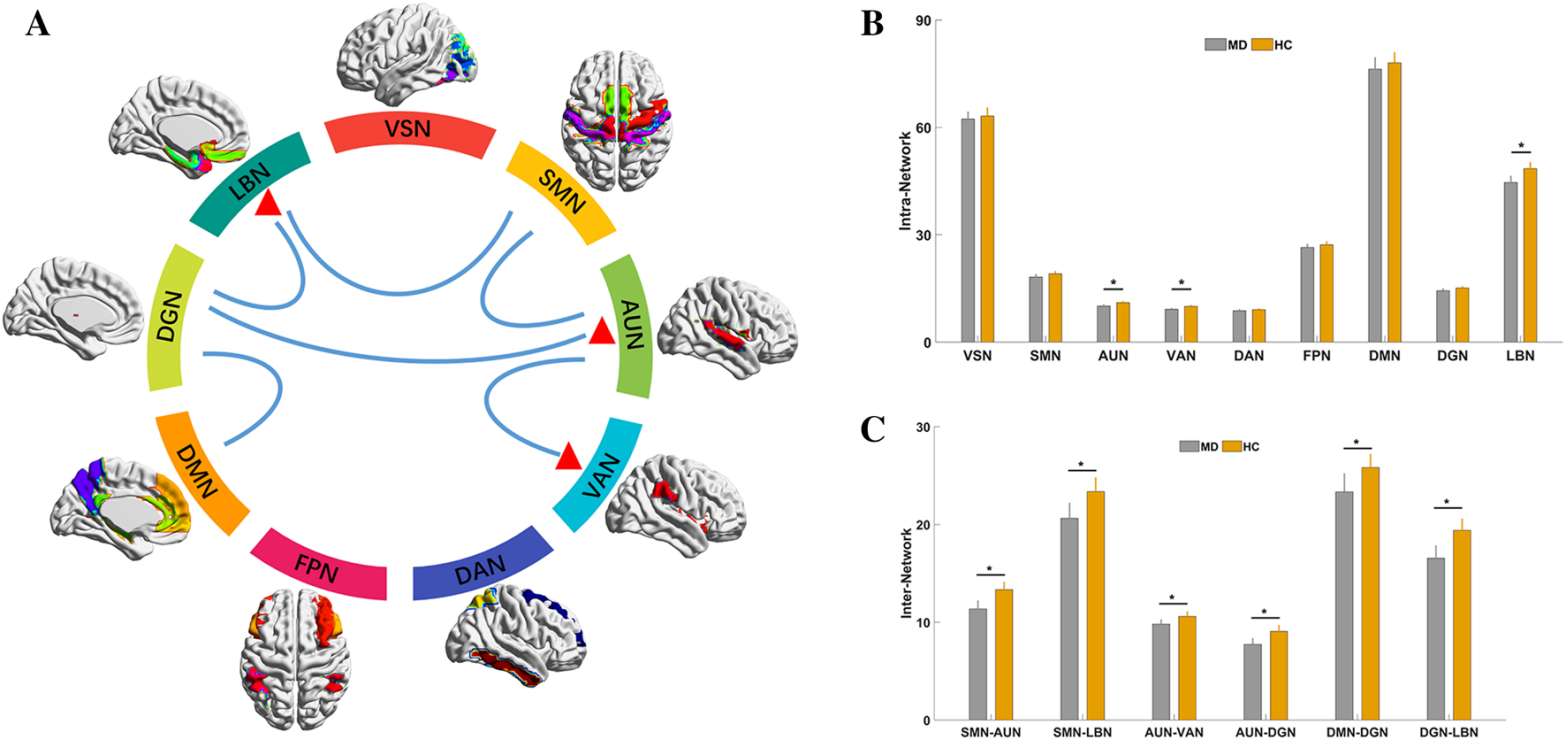
Altered functional connectivity among nine brain networks at intranetwork and internetwork level between MD and HC. (**A**) A colorful circle indicates the composite of nine brain networks. The red triangles indicate that the AUN, VAN and LBN showed altered intranetwork connections. The line linking two brain networks indicates altered internetwork connections. (**B**) The histogram shows the comparison between MD and HC of intranetwork connections of the nine brain networks. (**C**) The histogram shows the comparison between MD and HC of internetwork connections. *VSN* visual network, *SMN* somatomotor network, *AUN* auditory network, *VAN* ventral attention network, *DAN* dorsal attention network, *FPN* frontoparietal network, *DMN* default mode network, *DGN*, deep gray matter network, *LBN*, limbic network, *MD*, Ménière’s disease, *HC*, healthy control.

Compared to HC, patients with MD showed lower intranetwork connectivity strength in AUN (*p* = 0.035, Bonferroni corrected), VAN (*p* = 0.041, Bonferroni corrected), and LBN (*p* = 0.031, Bonferroni corrected). Meanwhile, MD patients showed lower internetwork connectivity strength in six pair subnetworks, including the AUN-SMN (*p* < 0.001, FDR corrected), AUN-VAN (*p* = 0.035, FDR corrected), AUN-DGN (*p* = 0.005, FDR corrected), DGN-DMN (*p* = 0.034, FDR corrected), DGN-LBN (*p* = 0.002, FDR corrected), and SMN-LBN (*p* = 0.013, FDR corrected) (Fig. 2A–C).

### Edge level: altered functional connectivity across ROI pairs in patients with MD

**Figure 3:**
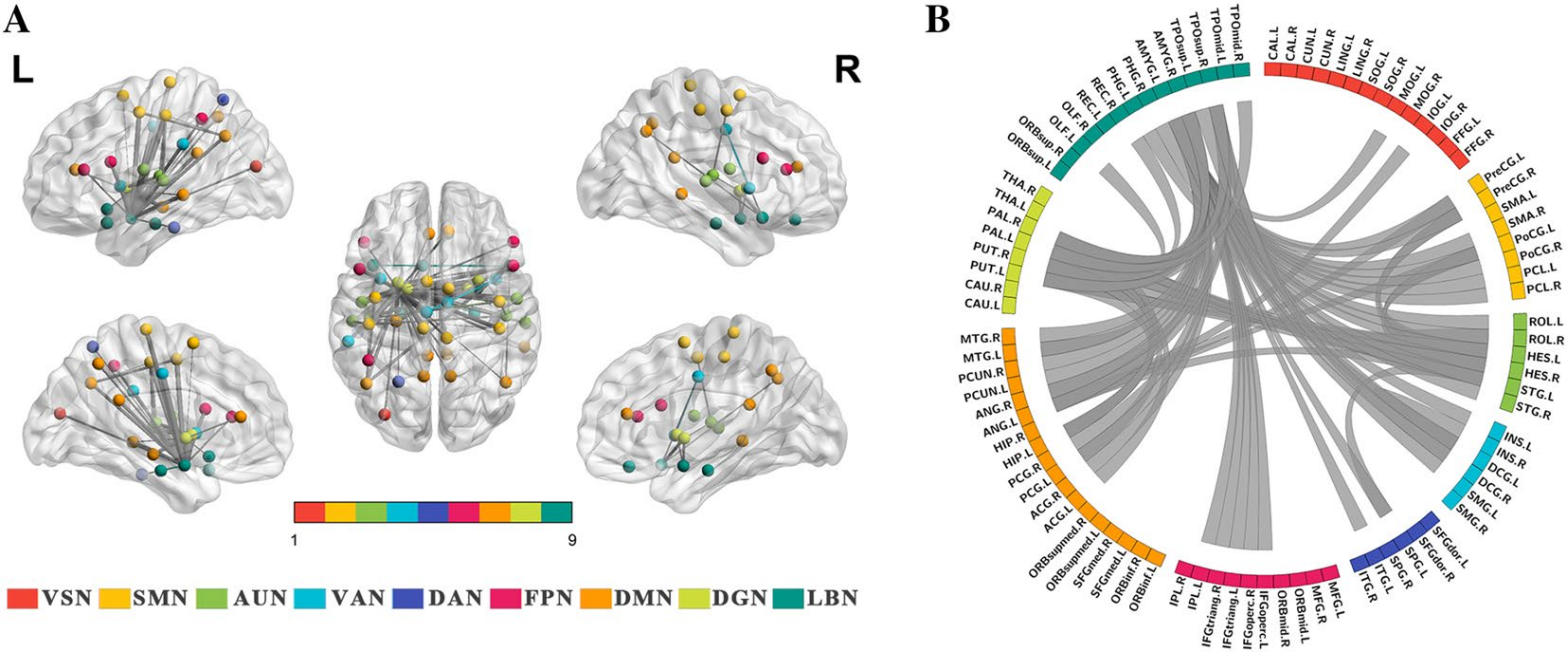
Altered functional connectivity of nodes at the edge level between MD and HC. (**A**) Brain surface rendering displaying altered functional connectivity (*p* < 0.05, FDR corrected) between MD and HC. The same color of spheres represented areas were from the same brain network. The thickness of the lines corresponds to the T values of the connectivity strength. (**B**) Circular representation illustrating altered functional connectivity patterns between MD and HC. *VSN* visual network, *SMN* somatomotor network, *AUN* auditory network, *VAN* ventral attention network, *DAN* dorsal attention network, *FPN* frontoparietal network, *DMN* default mode network, *DGN* deep gray matter network, *LBN*, limbic network.

At the edge level, after controlling for age, sex, and education, patients with MD compared with HC showed lower functional connectivity in 81 ROI pairs (all *p* < 0.05, FDR corrected), accounting for 2.02% of the 4005 analyzed ROI pairs. Among these, 0.90% of the pairs were intra-network, and 1.85% were internetwork pairs. The altered functional connectivity mainly involved the brain networks SMN, AUN, VAN, DMN, and LBN (Fig. 3).

### Correlation analysis

**Figure 4:**
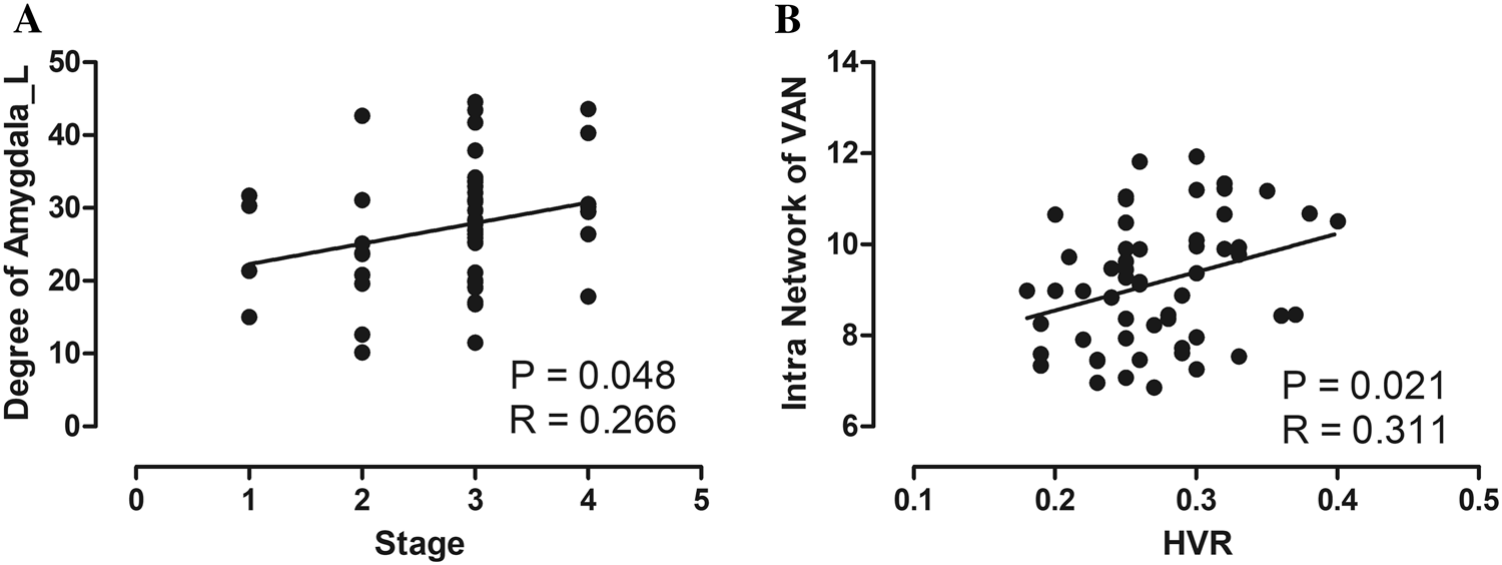
Correlations between altered brain network index and clinical information. (**A**) Correlations between the stage of MD and the degree of the left amygdala. (**B**) Correlations between the VR scores on the healthy side and the intranetwork connections of the VAN. *MD* Ménière’s disease, *VAN* ventral attention network, *HVR* vestibular hydrops ratio of healthy side.

We found a significant positive correlation between the severity of MD and the degree of the left amygdala (*p* = 0.048, R = 0.266). Moreover, a significant positive correlation was observed between the VR scores on the healthy side and the intranetwork connections of the VAN (Fig. 4A,B).

## Discussion

The present study employed rs-fMRI to investigate the patterns of functional reorganization in intranetwork and internetwork connectivity between individuals with MD and HC. This multi-level analysis explored connectivity integrity, network-level alterations, and specific connections at the edge level to provide a comprehensive understanding of functional connectivity changes in MD. One of the most intriguing findings of this study pertains to the widespread reductions in functional connectivity degree across multiple brain areas encompassing various networks, such as the SMN, AUN, VAN, DMN, LBN, and DGN. These findings suggest a broad disruption in the integration of neural activity within these networks in MD. Furthermore, we observed lower intranetwork connectivity within the AUN, VAN, and LBN, indicating impaired communication between regions involved in auditory processing, attentional control, and emotional regulation. Additionally, we noted lower internetwork connectivity between the SMN and LBN, and between the AUN and SMN, DGN, and VAN, and between the DGN and LBN, DMN. These internetwork connectivity alterations suggest disrupted coordination and information exchange between critical functional networks involved in motor control, emotional processing, and attentional mechanisms. Taken together, these findings provide novel insight into functional organization patterns within and between functional brain networks in MD.

The alterations observed in the auditory network provide important insights into the underlying mechanisms of auditory symptoms in MD. Our findings revealed functional connectivity degree was lower in MD compared to HC within the auditory network, indicating disrupted integration of neural activity among regions involved in auditory processing^28,29^. These findings align with the characteristic symptom of hearing loss experienced by individuals with MD. The observed reductions in connectivity within the auditory network may reflect impaired communication between auditory processing regions, potentially contributing to the deficits in auditory perception and discrimination observed in MD patients^1,2^. The auditory network alterations observed in our study may arise from both peripheral and central mechanisms. Peripheral vestibular dysfunction in MD, especially EH, may directly impact the cochlear and vestibular systems, leading to alterations in the auditory processing pathways^30^. Additionally, central mechanisms involving changes in neural plasticity and compensatory processes could contribute to the observed alterations in the auditory network^20,21^. The disrupted connectivity within the auditory network may interact with and influence other symptoms associated with MD, such as tinnitus and cognitive impairments^25,31^.Our study expands on these findings by revealing additional alterations within the auditory network and shedding light on the broader functional connectivity disruptions in MD. The observed alterations in the auditory network connectivity have potential clinical implications. They may serve as objective biomarkers for diagnosing and monitoring the progression of MD. Future studies could explore the relationship between the severity of auditory symptoms and the degree of connectivity changes within the auditory network. Additionally, investigating the effects of interventions targeted at restoring normal auditory network connectivity could provide valuable insights into potential treatment strategies for MD.

The alterations observed in the SMN, VAN, LBN, and DGN provide valuable insights into the functional disruptions associated with MD. Our findings revealed that functional connectivity degree within these networks was lower in MD compared to HC, indicating compromised integration of neural activity among regions involved in motor control, attentional processing, emotional regulation, and deep gray matter structures^11,22,25^. These alterations may contribute to the motor, attentional, emotional, and autonomic symptoms experienced by individuals with MD.

The lower connectivity degree within the SMN suggests impaired integration of neural activity among motor planning and execution regions. These findings align with the characteristic motor symptoms, including balance impairments and motor coordination difficulties, commonly observed in MD^1,2^. The alterations within the VAN indicate disrupted communication between regions involved in attentional control and salience detection. These changes may underlie attentional disturbances reported by MD patients, such as difficulties in focusing and maintaining attention^1,2^.

The observed alterations within the LBN are consistent with the emotional symptoms frequently associated with MD. The LBN plays a crucial role in emotional regulation and processing, and the lower connectivity within this network may contribute to the increased prevalence of anxiety and depression^22,25^. Additionally, the connectivity changes within the deep gray matter structures, such as the basal ganglia and thalamus, may impact motor control, emotional regulation, and autonomic functions^32^. These alterations could contribute to the diverse symptoms experienced in MD, including emotional instability, autonomic dysfunction, and visceral symptoms.

Moreover, we identified 81 pairs of brain areas exhibiting significant differences in functional connectivity between MD patients and healthy controls (HC) at the edge level. These specific connections may represent critical communication pathways affected by MD and further contribute to our understanding of the disease’s pathophysiology. Furthermore, our results demonstrated a positive correlation between the left amygdala’s functional connectivity degree and MD disease stage, suggesting that the amygdala’s involvement in emotional processing and stress regulation may play a role in disease progression. Additionally, the intranetwork connectivity within the ventral attention network was positively correlated with the healthy side vestibular response, indicating that the functional integrity of this network may be associated with the compensatory mechanisms observed in some MD patients.

It is important to acknowledge several limitations of our study. First, the cross-sectional design precludes us from drawing definitive conclusions about causality or the temporal dynamics of functional connectivity changes. Longitudinal studies would be valuable in elucidating the progressive nature of the observed alterations. Second, our sample size was relatively small, and future studies with larger cohorts are needed to validate these findings. Third, it is important to note that in this study, a 12-channel head coil was used for MRI acquisition, which may potentially lead to limitations in image spatial resolution and sensitivity. While a 12-channel head coil represents the minimum number of channels typically employed for head imaging, this choice was made based on the available equipment at our institution and its suitability for the study objectives. Finally, we acknowledge that comorbidities, medications, and other factors may have influenced our results, and further investigations controlling for these variables are warranted.

In conclusion, our study provides evidence of functional reorganization in multiple brain networks in MD. These findings expand our understanding of the neural mechanisms underlying the disease and shed light on the functional implications of altered connectivity patterns. The identified connections and correlations with disease stage and vestibular response contribute to the potential development of biomarkers and therapeutic targets for MD. Future research should focus on longitudinal investigations with larger cohorts to validate these findings and explore the clinical implications of the observed alterations in functional connectivity.

## Methods

### Subjects

Fifty-five patients with MD and seventy healthy control (HC) subjects were recruited from Wuhan Union Hospital. Only adult participants aged 18 years and older were included in the study. The diagnosis of MD was established following the diagnostic guidelines of MD outlined by the Bárány Society^33^. Exclusion criteria included: (1) patients fulfilling both diagnostic criteria of vestibular migraine and MD; (2) middle or inner ear infections (otitis media, mastoiditis, labyrinthitis etc.); (3) middle or inner ear anomaly (common cavity malformation, semicircular canal dysplasia, enlarged vestibular aqueduct, etc.); (4) bilateral MD; (5) having received previous ear surgery or intratympanic injections; (6) retro-cochlear lesions (vestibular schwannoma, internal acoustic canal stenosis etc.); (7) head trauma. Inclusion criteria for HCs encompassed the following aspects: (1) the absence of any known neurological or psychiatric disorders; (2) no history of hearing impairment or vestibular disorders; (3) normal audiometric findings, including pure-tone audiometry and tympanometry within specified parameters; (4) no contraindications for MRI scans. Exclusion criteria for HCs were defined as the presence of any abnormal structural manifestations, such as stroke or brain tumor, on magnetic resonance images, as well as the presence of white matter lesions exceeding 1 mm in size, visible on structural MRI.

This study was conducted in compliance with the latest version of the Declaration of Helsinki and was approved by the Medical Ethics Committee of Tongji Medical College of Huazhong University of Science and Technology (2020IEC-J330). All participants provided written informed consent after receiving a detailed explanation of the study’s purpose and procedures.

### Audio-vestibular evaluations

The detailed procedures of audio-vestibular evaluations have been described in our previous studies^34^, which included the pure tone audiometry, electrocochleogram (ECochG), glycerol test, caloric test. The audio-vestibular tests were performed within an interval of 3 days.

Pure-tone audiometry was conducted in a soundproof room, at 0.125, 0.25, 0.5, 1.0, 2.0, 4.0 and 8.0 kHz. The clinical stage of MD was determined based on three-frequency pure tone average (PTA), calculated as simple arithmetic means of 0.5, 1.0, and 2.0 kHz pure tone threshold. PTA of < 26 dB hearing loss (HL) was classified as Stage I; PTA of 26–40 dB HL as Stage II; PTA of 41–70 dB HL as Stage III; and PTA of > 70 dB HL as Stage IV.

Summating potential (SP) and action potential (AP) were recorded during ECochG. A positive ECochG result was defined as SP/ AP ratio greater than 0.4, which indicated the presence of EH. In glycerol test, two patterns of pathological results were noted, i.e., glycerol-induced hearing gain (improvement of hearing ability after glycerol intake) and rebound phenomena (deterioration of hearing ability after glycerol intake). The result of audiometric glycerol test was deemed positive when the pure-tone hearing ability was improved by: (1) ≥ 10 dB at any three or more frequencies or (2) ≥ 15 dB at one frequency at any time point after glycerol ingestion.

Bithermal caloric response was measured using infrared videonystagmography (Visual Eyes VNG, Micromedical Technologies, Chatham, IL). Patients lay supine with their upper trunk elevated at 30°. Cold (24 °C) and warm (50 °C) air stimulation was delivered into each external auditory canal alternately, and the maximum slow phase velocity (SPV_max_) of the caloric nystagmus was measured after each irrigation. The value of canal paresis (CP) was calculated using the Jongkees formula. Interaural asymmetry of the caloric nystagmus ≥ 25% was taken as evidence of unilateral vestibular hypofunction. Bilateral vestibular hypofunction is considered if SPV_max_ of each ear was less than 6°/s after caloric stimulation, or the summated SPVmax was less than 20°/s for all four stimulation conditions.

### Magnetic resonance imaging acquisition

All participants were scanned on a 3.0 Tesla MRI scanner (MAGNETOM trio; Siemens Healthcare, Erlangen, Germany) using a 12-channel head coil. All subjects were instructed to lie supine and keep still, with headphones covering their ears to reduce scanner noise. Head motion was minimized using a foam cushion. Anatomical images were acquired with a 3D high-resolution T1-weighted magnetization-prepared rapid acquisition gradient echo (MP-RAGE) sequence, with the following parameters: repetition time (TR) = 2250 ms, echo time (TE) = 2.26 ms, inversion time (TI) = 900 ms, flip angle = 9°, voxel size = 1.0 × 1.0 × 1.0 mm^3^, field of view (FOV) = 256 mm × 256 mm, slice thickness = 1.00 mm, and 176 sagittal slices covering the entire brain. Functional images were acquired using a gradient echo type echo planar imaging (EPI) sequence, with TR = 2000 ms, TE = 30 ms, flip angle = 90°, voxel size = 3 × 3 × 3 mm^3^, and FOV = 200 mm × 200 mm, resulting in 240 functional images. A T2-weighted sequence was also acquired to evaluate the peripheral auditory system, with the following parameters: TR = 1000 ms, TE = 132 ms, slice thickness = 0.5 mm, slice number = 64, flip angle = 120°, FOV = 200 mm × 200 mm, and averages = 2. Two radiologists independently reviewed the MR images (1 with 10 years of experience and one with more than 20 years of experience). Subjects with MR abnormalities, such as otitis media, acoustic neuroma, and brain tumors, were excluded from the analysis. Intratympanic Gd injection and MRI examination was performed as previously described^34^. The Gd-DTPA-dimeglumine solution (MultiHance; Braccosine, Shanghai, China) was used as the contrast agent. Delayed 3D-FLAIR MRI was used to evaluated the degree of EH. According to the published criteria^35^, the degree of EH in the vestibule and cochlea was classified into 3 grades: none, mild, and significant (Table 2). The vestibular hydrops ratio (VR) represents the proportion of the endolymphatic space area in relation to the entire lymphatic space in the vestibule. It was calculated using the following equation: VR = (number of negative pixels for the endolymph in the region of interest (ROI)/total number of pixels in the ROI).

**Table 2:** Grading of EH using magnetic resonance imaging.

Grade of hydrops	Vestibule (area ratio)	Cochlea
None	≤ 33.3%	No displacement of Reissner’s membrane
Mild	> 33.3%, ≤ 50%	Displacement of Reissner’s membrane Area of cochlear duct ≤ area of the scala vestibuli
Significant	> 50%	Area of cochlear duct exceeds the area of the scala vestibuli

*EH* endolymphatic hydrops.

### Magnetic resonance imaging data processing

Functional MRI (fMRI) images were preprocessed using the Data Processing and Analysis for Brain Imaging (DPABI) software (http://rfmri.org/dpabi) toolbox^36^ which is based on the Statistical Parametric Mapping (SPM12) toolkits (http://www.fil.ion.ucl.ac.uk/spm/software/spm12/) on the Matlab (R2017a) platform. The first 10 volumes of the resting-state blood oxygen level-dependent (BOLD) data were discarded to account for T1 equilibration effects, leaving 230 time points for analysis. Slice timing and motion correction were performed on the remaining volumes using a six-parameter rigid-body transformation. Nuisance signals, including linear trends, white matter signal, cerebrospinal fluid signal, and Friston 24 head motion parameters, were regressed out from the functional signal. The functional images were then normalized to the Montreal Neurological Institute (MNI) template using the Diffeomorphic Anatomical Registration Through Exponentiated Lie algebra (DARTEL) tool, after being coregistered with the corresponding structural images, which were also normalized to the MNI template. Finally, the images were smoothed with a 4 mm full width at half-maximum Gaussian kernel and band-pass filtered with a frequency range of 0.01–0.1 Hz. Participants with a maximum head motion greater than 1.5 mm in displacement or 1.5° in rotation, as well as those with a mean frame-wise displacement (FD) calculated by the Jenkinson method greater than 0.2, were excluded from the study.

### Functional connectivity analyses

To examine the functional connectivity patterns of the various brain networks, we used the 90 Automated Anatomical Labeling (AAL) regions^37^, which were clustered into seven networks, including visual (VSN), somatomotor (SMN), dorsal attention (DAN), ventral attention (VAN), limbic (LBN), frontoparietal (FPN), and default mode (DMN) networks, based on previous studies^26,38,39^. Furthermore, the bilateral caudate, putamen, pallidum, and thalamus were identified and clustered as the deep gray matter network (DGN)^26,39,40^. As the auditory network (AUN) plays a unique role in MD disease, we separated it from the SMN network for subsequent analysis. Thus, in our subsequent analysis, we investigated the patterns of functional connectivity among these nine brain subnetworks.

To quantify the functional connectivity between brain regions, we calculated Pearson’s correlation coefficients (r values) between the mean times of each pair of regions, resulting in a 90 × 90 correlation matrix for each subject. We then converted the correlation coefficients to nodal connectivity degree η using an exponential function, as described in previous studies^26,27^. We assessed intranetwork and internetwork connections by averaging the transformed correlation coefficients of all ROI pairs within or between each particular brain network^26,27,39,40^.

We performed functional connectivity analyses at the integrity, network, and edge levels to study the shared general and distinct specific connectivity patterns between the MD and HC groups^26,27,39^. At the integrity level, we investigated the information flow received from specific nodes, which is equivalent to the “degree centrality” in graph theory^27^. At the network level, we performed intranetwork and internetwork analysis. Intranetwork analysis measured connectivity by averaging the transformed correlation coefficients within a network, while internetwork analysis measured the connectivity between two networks^41^. At the edge level, we explored the functional connectivity between all possible node pairs. This approach allowed us to accurately study connectivity between nodes and reveal focal phenomena obscured by the integration of connected brain regions as networks^41^.

### Statistical analysis

For the demographic data, data processing and statistical analysis were conducted using SPSS 17.0 software (SPSS Inc., Chicago, IL, USA). Continuous variables were reported as medians and interquartile intervals, while categorical variables were presented as frequencies and proportions.

To test for group differences in functional connectivity, we conducted independent samples t-tests on the Z scores for each level (integrity, network, and edge) between the MD and HC groups, with age, gender, and education level included as covariates in a general linear model. Statistical significance was set at a corrected *p*-value of < 0.05, using a permutation-based method. In addition, we performed multiple regression analyses to examine the relationship between functional connectivity and the clinical indices in MD patients, including parameters such as MD duration, MD stages, among others, while controlling for covariates such as age, gender, and education level.

## Data Availability

The data that support the findings of this study are available from the corresponding author upon reasonable request.

## References

[1] Nakashima T (2016). Meniere’s disease. Nat. Rev. Dis. Primers.

[2] Rizk HG (2022). Pathogenesis and etiology of meniere disease: a scoping review of a century of evidence. JAMA Otolaryngol. Head Neck Surg..

[3] Yang CH, Yang MY, Hwang CF, Lien KH (2023). Functional and molecular markers for hearing loss and vertigo attacks in Meniere’s disease. Int. J. Mol. Sci..

[4] Harris JP, Alexander TH (2010). Current-day prevalence of Meniere’s syndrome. Audiol. Neurootol..

[5] Merchant SN, Adams JC, Nadol JB (2005). Pathophysiology of Meniere’s syndrome: Are symptoms caused by endolymphatic hydrops?. Otol. Neurotol..

[6] Nakashima T (2007). Visualization of endolymphatic hydrops in patients with Meniere’s disease. Laryngoscope.

[7] Seo YJ, Kim J, Kim SH (2016). The change of hippocampal volume and its relevance with inner ear function in Meniere’s disease patients. Auris. Nasus. Larynx.

[8] Jian H (2023). Effect of late-stage meniere’s disease and vestibular functional impairment on hippocampal atrophy. Laryngoscope.

[9] Yuan X (2022). Microstructural changes of the vestibulocochlear nerve in patients with Meniere’s disease using diffusion tensor imaging. Front. Neurol..

[10] Chai Y (2023). Functional connectomics in depression: Insights into therapies. Trends Cogn. Sci..

[11] Sporns O, Betzel RF (2016). Modular brain networks. Annu. Rev. Psychol..

[12] Fox MD, Raichle ME (2007). Spontaneous fluctuations in brain activity observed with functional magnetic resonance imaging. Nat. Rev. Neurosci..

[13] Van Ombergen A (2017). Altered functional brain connectivity in patients with visually induced dizziness. Neuroimage Clin..

[14] Lee JO (2018). Altered brain function in persistent postural perceptual dizziness: A study on resting state functional connectivity. Hum. Brain Mapp..

[15] Li K (2020). Altered spontaneous functional activity of the right precuneus and cuneus in patients with persistent postural-perceptual dizziness. Brain Imaging Behav..

[16] Li K (2020). Altered intra- and inter-network functional connectivity in patients with persistent postural-perceptual dizziness. Neuroimage. Clin..

[17] Wang S (2019). Alterations of structural and functional connectivity in profound sensorineural hearing loss infants within an early sensitive period: A combined DTI and fMRI study. Dev. Cogn. Neurosci..

[18] Cui W, Wang S, Chen B, Fan G (2021). Altered functional network in infants with profound bilateral congenital sensorineural hearing loss: A graph theory analysis. Front. Neurosci..

[19] Guo P (2021). Alterations of regional homogeneity in children with congenital sensorineural hearing loss: A resting-state fMRI study. Front. Neurosci..

[20] Xu H (2016). Disrupted functional brain connectome in unilateral sudden sensorineural hearing loss. Hear Res..

[21] Hua JC (2022). Aberrant functional network of small-world in sudden sensorineural hearing loss with tinnitus. Front Neurosci.

[22] Leaver AM, Seydell-Greenwald A, Rauschecker JP (2016). Auditory-limbic interactions in chronic tinnitus: Challenges for neuroimaging research. Hear Res..

[23] Gentil A (2019). Alterations in regional homogeneity in patients with unilateral chronic tinnitus. Trends Hear.

[24] Job A, Jaroszynski C, Kavounoudias A, Jaillard A, Delon-Martin C (2020). Functional connectivity in chronic nonbothersome tinnitus following acoustic trauma: A seed-based resting-state functional magnetic resonance imaging study. Brain Connect..

[25] Husain FT, Schmidt SA (2014). Using resting state functional connectivity to unravel networks of tinnitus. Hear Res..

[26] Zeng W (2022). Altered intra- and inter-network connectivity in drug-naive patients with early parkinson’s disease. Front. Aging Neurosci..

[27] Chen J (2016). Convergent and divergent intranetwork and internetwork connectivity patterns in patients with remitted late-life depression and amnestic mild cognitive impairment. Cortex.

[28] Lumaca M, Kleber B, Brattico E, Vuust P, Baggio G (2019). Functional connectivity in human auditory networks and the origins of variation in the transmission of musical systems. Elife.

[29] Wei Z (2021). Reorganization of auditory-visual network interactions in long-term unilateral postlingual hearing loss. J. Clin. Neurosci..

[30] Badash I (2021). Endolymphatic hydrops is a marker of synaptopathy following traumatic noise exposure. Front. Cell Dev. Biol..

[31] Kok TE (2022). Resting-state networks in tinnitus: A scoping review. Clin. Neuroradiol..

[32] Pierce JE, Peron J (2020). The basal ganglia and the cerebellum in human emotion. Soc. Cogn. Affect Neurosci..

[33] Lopez-Escamez JA (2015). Diagnostic criteria for Meniere’s disease. J. Vestib. Res..

[34] Leng Y (2023). Comparison between audio-vestibular findings and contrast-enhanced MRI of inner ear in patients with unilateral Meniere’s disease. Front. Neurosci..

[35] Nakashima T (2009). Grading of endolymphatic hydrops using magnetic resonance imaging. Acta Otolaryngol. Suppl..

[36] Yan CG, Wang XD, Zuo XN, Zang YF (2016). DPABI: Data processing & analysis for (Resting-State) brain imaging. Neuroinformatics.

[37] Tzourio-Mazoyer N (2002). Automated anatomical labeling of activations in SPM using a macroscopic anatomical parcellation of the MNI MRI single-subject brain. Neuroimage.

[38] Yeo BT (2011). The organization of the human cerebral cortex estimated by intrinsic functional connectivity. J. Neurophysiol..

[39] Liu J (2022). Altered brain network in first-episode, drug-naive patients with major depressive disorder. J. Affect. Disord..

[40] Long X (2020). Altered brain white matter connectome in children and adolescents with prenatal alcohol exposure. Brain Struct. Funct..

[41] Brier MR (2012). Loss of intranetwork and internetwork resting state functional connections with Alzheimer’s disease progression. J. Neurosci..

